# A Microphysiological System for Studying Nonalcoholic Steatohepatitis

**DOI:** 10.1002/hep4.1450

**Published:** 2019-11-13

**Authors:** Tomasz Kostrzewski, Paloma Maraver, Larissa Ouro‐Gnao, Ana Levi, Sophie Snow, Alina Miedzik, Krista Rombouts, David Hughes

**Affiliations:** ^1^ CN Bio Innovations Ltd. Welwyn Garden City Hertfordshire United Kingdom; ^2^ Institute for Liver and Digestive Health, Regenerative Medicine and Fibrosis Group, Royal Free University College London United Kingdom

## Abstract

Nonalcoholic steatohepatitis (NASH) is the most severe form of nonalcoholic fatty liver disease (NAFLD), which to date has no approved drug treatments. There is an urgent need for better understanding of the genetic and molecular pathways that underlie NAFLD/NASH, and currently available preclinical models, be they *in vivo* or *in vitro*, do not fully represent key aspects of the human disease state. We have developed a human *in vitro* co‐culture NASH model using primary human hepatocytes, Kupffer cells and hepatic stellate cells, which are cultured together as microtissues in a perfused three‐dimensional microphysiological system (MPS). The microtissues were cultured in medium containing free fatty acids for at least 2 weeks, to induce a NASH‐like phenotype. The co‐culture microtissues within the MPS display a NASH‐like phenotype, showing key features of the disease including hepatic fat accumulation, the production of an inflammatory milieu, and the expression of profibrotic markers. Addition of lipopolysaccharide resulted in a more pro‐inflammatory milieu. In the model, obeticholic acid ameliorated the NASH phenotype. Microtissues were formed from both wild‐type and patatin‐like phospholipase domain containing 3 (PNPLA3) I148M mutant hepatic stellate cells. Stellate cells carrying the mutation enhanced the overall disease state of the model and in particular produced a more pro‐inflammatory milieu. *Conclusion:* The MPS model displays a phenotype akin to advanced NAFLD or NASH and has utility as a tool for exploring mechanisms underlying the disease. Furthermore, we demonstrate that in co‐culture the PNPLA3 I148M mutation alone can cause hepatic stellate cells to enhance the overall NASH disease phenotype.

## Graphical abstract

We have developed an advanced human *in vitro* co‐culture model of nonalcoholic steatohepatitis. The model was used to explore effects of genetic mutations in the PNPLA3 gene on hepatic stellate cell function and disease progression.

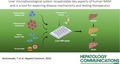

**Abbreviations**
3D: three‐dimensional
CXCL: chemokine (C‐X‐C motif) ligand
ELISA: enzyme‐linked immunosorbent assay
FFA: free fatty acid
GAPDH: glyceraldehyde 3‐phosphate dehydrogenase
HK: human Kupffer cell
HSC: hepatic stellate cell
IL: interleukin
LPS: lipopolysaccharide
MCP1: monocyte chemoattractant protein 1
MPS: microphysiological system
NAFLD: nonalcoholic fatty liver disease
NASH: nonalcoholic steatohepatitis
OCA: obeticholic acid
PCR: polymerase chain reaction
PHH: primary human hepatocyte
SNP: single nucleotide polymorphism
TGF‐β: transforming growth factor β
TIMP‐1: tissue inhibitor of metalloproteinase 1
TNF‐α: tumor necrosis factor α
WT: wild type

As a result of the increased prevalence of diabetes, obesity and metabolic syndrome, nonalcoholic fatty liver disease (NAFLD) is now the most common chronic liver disease in developed countries.^1^ NAFLD is a spectrum of pathologies ranging from benign hepatic steatosis through to nonalcoholic steatohepatitis (NASH), which can ultimately lead to cirrhosis and liver cancer. NASH is a serious condition, defined as a combination of hepatic steatosis, inflammation, hepatic damage, and pericellular liver fibrosis.^2^ The genetic basis of NAFLD has started to be explored, and the I148M mutation in the patatin‐like phospholipase domain containing 3 (*PNPLA3*) gene has been identified as the major genetic variant that associates with NAFLD/NASH progression.^3^ This single nucleotide polymorphism (SNP) can lead to more than 2‐fold increases in hepatic fat content in NAFLD patients^3^ and confers an up to a 10‐fold increased risk of liver cancer related to NAFLD.^4^ Despite the well‐established public health impact of NAFLD/NASH, there remains no approved therapy for the treatment of this disease.^2^ Significant numbers of compounds are now entering clinical development aimed at treating NAFLD/NASH, using a wide range of therapeutic approaches and targeting a diverse set of molecular pathways.

Our understanding of the molecular basis of NAFLD, and its development into NASH, has been based primarily on the use of rodent models (chemical, genetic, or diet‐induced).^5^, ^6^ Many preclinical rodent models have now been developed, but no individual model fully recapitulates the human disease and significant differences exist in the transcriptomic profile of the liver tissue, the manner in which triglycerides accumulate within the liver, and the level of hepatic fibrosis.^6^, ^7^, ^8^ There is no consensus on the best utility of these models, as evidenced by the more than 30 models that have now been developed.^6^, ^8^

Studying NASH using human *in vitro* models offers the ability to perform studies at the cellular level, allowing molecular mechanisms to be elucidated and the genetic drives of the disease to be specifically explored. Various approaches have been taken to study NAFLD/NASH using *in vitro* models, including the use of precision‐cut liver slices,^9^, ^10^ immortalized hepatic cell lines,^11^, ^12^, ^13^ and primary human hepatocytes.^14^, ^15^, ^16^ The culture of primary human cells *in vitro* represents the best opportunity to develop a model that can translate to the clinical disease, as these cells should most closely mimic the expression profiles and phenotypic features of the cells in patient livers. However, long‐term cultures (>1 week) of primary human hepatocytes (PHHs) are challenging, and only through recent technological advances (e.g., three‐dimensional [3D] spheroidal cultures, microfluidic perfusion, co‐cultures) has this become tractable.^17^ Therefore, to date, most *in vitro* NAFLD studies involving PHHs have focused on short‐term exposure (48‐72 hours) to free fatty acids (FFAs), allowing the study of transient responses to triglyceride challenge.^14^, ^15^, ^16^ Some studies using advanced *in vitro* platforms, including micropatterned co‐cultures of primary hepatocytes and murine fibroblasts,^18^ a hemodynamic co‐culture system^19^ or bioprinted cultures of primary liver cells,^20^ have started to demonstrate how exposure to glucose and FFAs can affect human hepatic cell types. Moreover, with the addition of transforming growth factor β (TGF‐β), early stages of fibrosis can be detected.^20^

We previously developed a model of hepatic steatosis using a 3D perfused microphysiological system (MPS), which enabled PHHs to be cultured for 2 weeks in the presence of FFAs, allowing the chronic effects of triglyceride accumulation to be analyzed.^21^ The perfused MPS maintains highly metabolically active PHHs for extended periods (up to 40 days)^22^, ^23^, ^24^ and has been demonstrated to support PHHs and human Kupffer cell (HK) co‐cultures to study the effects of liver inflammation on drug metabolism, drug–drug interactions, and liver toxicity.^22^, ^23^, ^24^

Here, we use the same MPS with a co‐culture of PHH, HK, and hepatic stellate cells (HSCs) to create a model of human NASH. These co‐cultures can be maintained under disease‐inducing conditions for at least 2 weeks and demonstrate key hallmarks of the human disease, including accumulation of intracellular triglyceride, the production of both pro‐inflammatory cytokines, and profibrotic markers. The model demonstrates a transcriptional profile that is consistent with a NASH phenotype and is susceptible to modulation with obeticholic acid (OCA), a compound in late‐stage clinical development for the treatment of NASH. Finally, we used the MPS NASH model to investigate how different genetic backgrounds can drive changes in the disease phenotype. In particular, we focused on the I148M mutation in the *PNPLA3* gene, the most strongly correlated SNP associated with NASH.^25^ We explored the effects of the I148M PNPLA3 mutation specifically in HSCs and demonstrated its impact on NASH disease progression. This highlights the mechanistic insights that can be gained through the modular nature of the *in vitro* model described here, and demonstrates the utility of the MPS as a tool for NASH drug discovery.

## Methods and Materials

### PHH Culture and Treatment

Cryopreserved PHHs and primary HKs were purchased from Life Technologies (Paisley, United Kingdom). Primary HSCs were obtained from the Institute for Liver and Digestive Health, Royal Free Hospital, University College London (London, United Kingdom). These cells had been isolated from resected liver wedges or livers unsuitable for transplant and were obtained from patients undergoing surgery at the Royal Free Hospital after providing informed consent (NC2015.020 B‐ERC‐RF). Cells were isolated as previously described.^26^ In total, nine HSC donors were used throughout the study (Supporting Table S1).

Cultures were performed in the LiverChip MPS (CN Bio Innovations Ltd., Welwyn Garden City, United Kingdom) (Supporting Fig. S1), either with PHH alone or in a co‐culture of PHH + HK + HSC. Use of the MPS has been described previously.^21^, ^22^ Briefly, PHHs were seeded at a density of 6 × 10^5^ viable cells with a potential for co‐culture with HKs and/or HSCs at a density of 6 × 10^4^ viable cells in 1.6 mL of medium per well and maintained under a flow rate of 1.0 µL/s. Cells were seeded in Williams E medium containing primary hepatocyte thawing and plating supplements (Life Technologies, Carlsbad, CA) for the first 24 hours of culture. Thereafter, cells were cultured in HEP‐LEAN or HEP‐FAT medium; both medias are proprietary to CN Bio Innovations. The HEP‐FAT medium is a derivative of the HEP‐LEAN medium and contains a mixture of saturated and unsaturated FFAs, as well as physiologically relevant quantities of insulin and sugars. Complete media changes were performed on all wells every 48 to 72 hours. Lipopolysaccharide (LPS) from Escherichia coli O111:B4 (Sigma Aldrich, St. Louis, MO) was added to the HEP‐FAT culture medium on day 8 of culture onward at a concentration of 0.5 ng/mL and maintained in the culture medium for the remainder of the experiment. Microtissues exposed to OCA (AdooQ Bioscience, Irvine, CA) were first cultured for 8 days in HEP‐FAT medium. OCA was dissolved in DMSO, diluted in cell culture medium (final DMSO concentration ≤0.5%), and added to microtissues during a full medium change. Cultures were maintained in HEP‐FAT medium in the presence of the compound for a further 7 days.

### Biomarker Profiling

Soluble biomarkers (e.g., interleukin [IL] 6, tumor necrosis factor α [TNF‐α], procollagen 1, tissue inhibitor of metalloproteinase 1 [TIMP‐1], fibronectin, albumin) in cell culture medium were analyzed using Quantikine and DuoSet enzyme‐linked immunosorbent assay (ELISA) kits (R&D Systems, Minneapolis, MN) and Luminex arrays. For the Bio‐Plex Pro Human group 1 analytes, 27‐plex analytes were combined with human chemokine Gro‐α/chemokine (C‐X‐C motif) ligand 1 (CXCL1) according to the manufacturer’s recommendations (Bio‐Rad Laboratories, Inc., Hercules, CA). Data from the samples were acquired using a Bio‐Rad MAGPIX Multiplex system. The software used for the analysis was xPONENT 4.2. Twelve analytes were excluded from the analysis, as they had no detectable expression or had no significant differences among any of the culture conditions. Oil Red O staining and FFA quantification were determined for samples from MPS cultures as previously described.^21^

### PNPLA3 I148M Genotyping

DNA was extracted from the primary hepatic cells of interest using DNazol (Life Technologies). The PNPLA3 rs738409 C→G SNP, encoding I148M, was genotyped using a Taqman assay (assay on demand for rs738409; Applied Biosystems, Foster City, CA). Post‐polymerase chain reaction (PCR) allelic discrimination was carried out on Applied Biosystems’ 7500/7500 real‐time PCR system.

### RNA Isolation and Gene‐Expression Analysis

Total RNA was extracted using TRIzol (Ambion, Inc., Austin, TX) and a chloroform phase separation and converted to complementary DNA. This was analyzed by Human Fatty Liver RT^2^ Profiler PCR Arrays (PAHS‐157Z) and Human Cytokines & Chemokines RT2 Profiler PCR Arrays (PAHS‐150Z) or using Taqman assays ACTA2 (Hs00426835_g1), IGFBP1 (Hs00236877_m1), IL‐6 (Hs00174131_m1), GK (Hs02340007_g1), CYP7A1 (Hs00167982_m1), CYGB (Hs00964894_g1), PCDH7 (Hs05574398_g1), Desmin (Hs00157258_m1), COL1A1 (Hs00164004_m1), and COL3A1 (Hs00943809_m1). Samples were analyzed using a Quantstudio 6 real‐time PCR system (Applied Biosystems). Ct values from samples were compared and normalized to house‐keeping gene expression.

### Statistics

All of the experiments were performed with at least three replicates, with cells obtained from three different PHH donors, if not indicated otherwise. Values reported are means ± SD, unless otherwise stated. Comparisons between groups were performed using Student *t* test. *P* values less than 0.05 were considered statistically significant.

## Results

**Figure 1:**
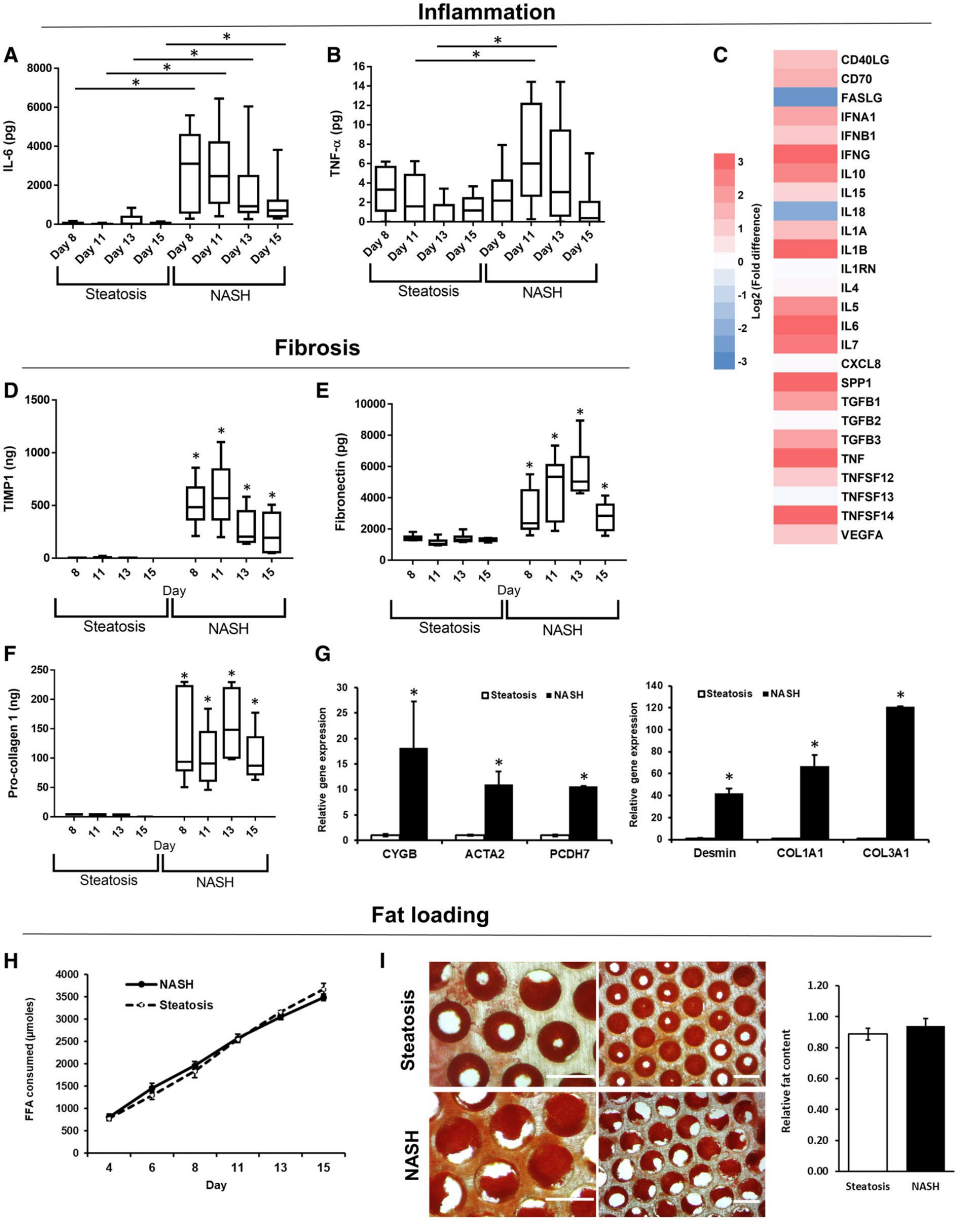
Phenotype of the *in vitro* human NASH model mimics key aspects of clinical disease. PHH alone (steatosis) or PHH, HK, and HSC co‐cultures (NASH) were cultured in the MPS for 15 days under high‐fat conditions (HEP‐FAT medium). Inflammatory responses were measured A) IL‐6 and B) TNF‐α production were measured by ELISA. (C) The expression of inflammatory/cytokine genes were analyzed in total RNA from both disease models using human cytokine RT2 profiler PCR arrays. Gene‐expression changes in the NASH model are expressed as a fold change over the steatosis model and are expressed as log2‐fold changes (red, up‐regulation; blue, down‐regulation). Fibrotic responses in the models were analyzed by determining the production of TIMP‐1 (D), fibronectin (E), and procollagen 1 (F) in cell culture medium, which were measured by ELISA. (G) Relative expression of profibrotic genes in total RNA, as measured by quantitative PCR; expression of each gene was normalized to glyceraldehyde 3‐phosphate dehydrogenase (GAPDH) and is shown as relative to steatosis samples. (H) Fat consumed by cultures over 15 days of culture was calculated by analyzing the culture medium for the presence of FFAs. (I) Fat loading in microtissues was confirmed using Oil Red O staining, which was quantified by absorbance at 510 nm; representative images also shown (scale bar = 400 µm). Data are expressed as means ± SD from a minimum of nine independent cultures (3 donors per condition and n = 3 per donor); *P < 0.05. Abbreviations: CD, clusters of differentiation; COL1A1, Collagen, type I, alpha 1; COL1A3, Collagen, type I, alpha 3; FASLG, Fas ligand; PCDH7, Protocadherin‐7; SPP1, Osteopontin; VEGFA, Vascular endothelial growth factor A.

We previously used a 3D perfused MPS to create a model of hepatic steatosis, consisting of a monoculture of PHHs cultured under high‐fat conditions.^21^ More advanced stages of NAFLD and NASH are defined by the presence of inflammation and fibrosis in the liver, and two nonparenchymal cell types known to drive these disease processes are HK and HSC.^2^ To generate an *in vitro* model of NASH, we co‐cultured primary human HKs and primary human HSCs together with PHHs in the MPS at the physiologically relevant ratio of 1:10.^27^ To compare the phenotype of the PHH, HK, and HSC co‐culture with the previously developed model of steatosis,^21^ both models were cultured for 15 days in HEP‐FAT medium, a hepatic cell culture medium with high levels of FFAs, and physiological levels of glucose and insulin. Both models were compared for a range of biomarkers expressed in NAFLD/NASH,^28^ and the NASH model was found to display a pro‐inflammatory phenotype with the detection of IL‐6 secretion (Fig. 1A), TNF‐α secretion (Fig. 1B), and the expression of a range of pro‐inflammatory genes (Fig. 1C). The NASH model also exhibited markers of fibrosis, with increased concentrations of TIMP‐1, fibronectin and procollagen 1, present in the culture medium (Fig. 1D‐F). The expression profiles of profibrotic genes such as *CYGB*, *ACTA2*, *PCDH7*, *DES*, *COL1A1,* and *COL1A3* were also significantly up‐regulated in the co‐culture NASH model, when compared with the steatosis model (Fig. 1G). Both models were observed to accumulate fat at a similar rate, had equivalent levels of intracellular fat loading (Fig. 1H‐J), and neither model was found to demonstrate any signs of hepatotoxicity (Supporting Fig. S2). However, the NASH model did produce slightly lower amounts of albumin (Supporting Fig. S2), matching observations from the clinic, where reduced albumin correlates with advanced liver disease.^28^, ^29^, ^30^ PHHs in the MPS form microtissues within the 3D collagen‐coated scaffold, and to visualize the distribution of HKs and HSCs within these microtissues, each cell type was transduced with an adenovirus expressing a fluorescent marker (Supporting Fig. S3). HKs and HSCs were found to integrate, essentially randomly, within the PHH microtissues, allowing direct interaction among the different cell types (Supporting Fig. S3).

To investigate more broadly the difference between the steatosis model and the co‐culture NASH model, the gene‐expression profiles of 84 genes associated with fatty liver disease were analyzed. These genes included those involved with key metabolic pathways and disease‐associated mechanisms (e.g., inflammation). When compared with control samples (PHHs alone in the absence of fat loading), the steatosis model only showed six genes with significantly altered expression (Table 1), similar to our previous observations,^21^ whereas 25 genes were differentially expressed in the NASH model (Table 1 and Supporting Fig. S4). Expression changes were observed in genes associated with insulin signaling (e.g., IGFBP1), cholesterol metabolism (e.g., CD36, PPARγ), glucose metabolism (e.g., G6PD), lipid metabolism (e.g., FABP5, LPL), and inflammation (e.g., IL‐6, TNF‐α) (Table 1). Together, these data demonstrated that the co‐culture NASH model has a distinct and different phenotype to the steatosis model with increased levels of inflammation, fibrosis markers, and an altered transcriptomic profile.

**Table 1:** Gene‐Expression Profiles Are Altered More Significantly in the *In Vitro* NASH Model Than Under Steatosis Conditions

Pathway/Gene	Steatosis		NASH	
	Fold Change	*P* Value	Fold Change	*P* Value
Insulin Signaling				
IGF1	1.58	0.31	3.72	<0.01
IGFBP1	2.65	0.28	41.91	0.002
mTOR	1.25	0.07	0.26	<0.01
PKLR	1.36	0.15	0.14	<0.01
SOCS3	1.52	0.32	292.60	<0.01
Glucose Metabolism				
G6PC	1.67	0.11	0.40	0.002
G6PDH	1.38	0.33	5.99	<0.01
GCK	1.46	0.34	0.10	<0.01
PDK4	4.33	0.02	1.56	0.086
Cholesterol Metabolism				
ABCA1	0.99	0.94	1.97	0.032
CD36	1.25	0.76	167.67	<0.01
CYP2E1	5.02	0.02	7.56	0.016
CYP7A1	5.24	0.01	0.14	<0.01
PPARG	1.24	0.48	3.20	0.006
Lipid Metabolism				
FABP3	1.70	0.33	6.73	<0.01
FABP5	1.40	0.14	13.42	<0.01
FASN	1.40	0.31	0.04	<0.01
GK	1.74	0.22	8.50	0.029
LPL	1.03	0.77	75.08	<0.01
SCD	1.06	0.90	0.16	<0.01
Inflammation				
CEBPB	1.46	0.08	0.55	0.019
IFN‐γ	1.27	0.11	4.38	<0.01
IL‐10	1.27	0.11	6.66	<0.01
IL‐1B	0.43	0.08	10.12	<0.01
IL‐6	0.69	0.87	481.35	<0.01
TNF	1.44	0.43	10.79	<0.01

Abbreviations: ABCA1, ATP‐Binding Cassette, Sub‐Family A, Member 1; CD36, clusters of differentiation 36; CEBPB, CCAAT Enhancer Binding Protein Beta; CYP2E1, Cytochrome P450 Family 2 Subfamily E Member 1; CYP7A1, Cytochrome P450 Family 7 Subfamily A Member 1; FABP, fatty acid‐binding protein; FASN, Fatty Acid Synthase; G6P, glucose 6‐phosphate; G6PDH, glucose‐6‐phosphate dehydrogenase; GCK, Glucokinase; GK, Glycerol Kinase; IFN‐γ, interferon gamma; IGF1, insulin‐like growth factor 1; IGFBP1, insulin‐like growth factor‐binding protein 1; LPL, Lipoprotein Lipase; mTOR, mammalian target of rapamycin; PDK4, pyruvate dehydrogenase kinase isozyme 4; PKLR, Pyruvate Kinase R‐Type/L‐Type; PPAR‐γ, peroxisome proliferator‐activated receptor gamma; SCD, Stearoyl‐CoA Desaturase; and SOCS3, suppressor of cytokine signaling 3.

**Figure 2:**
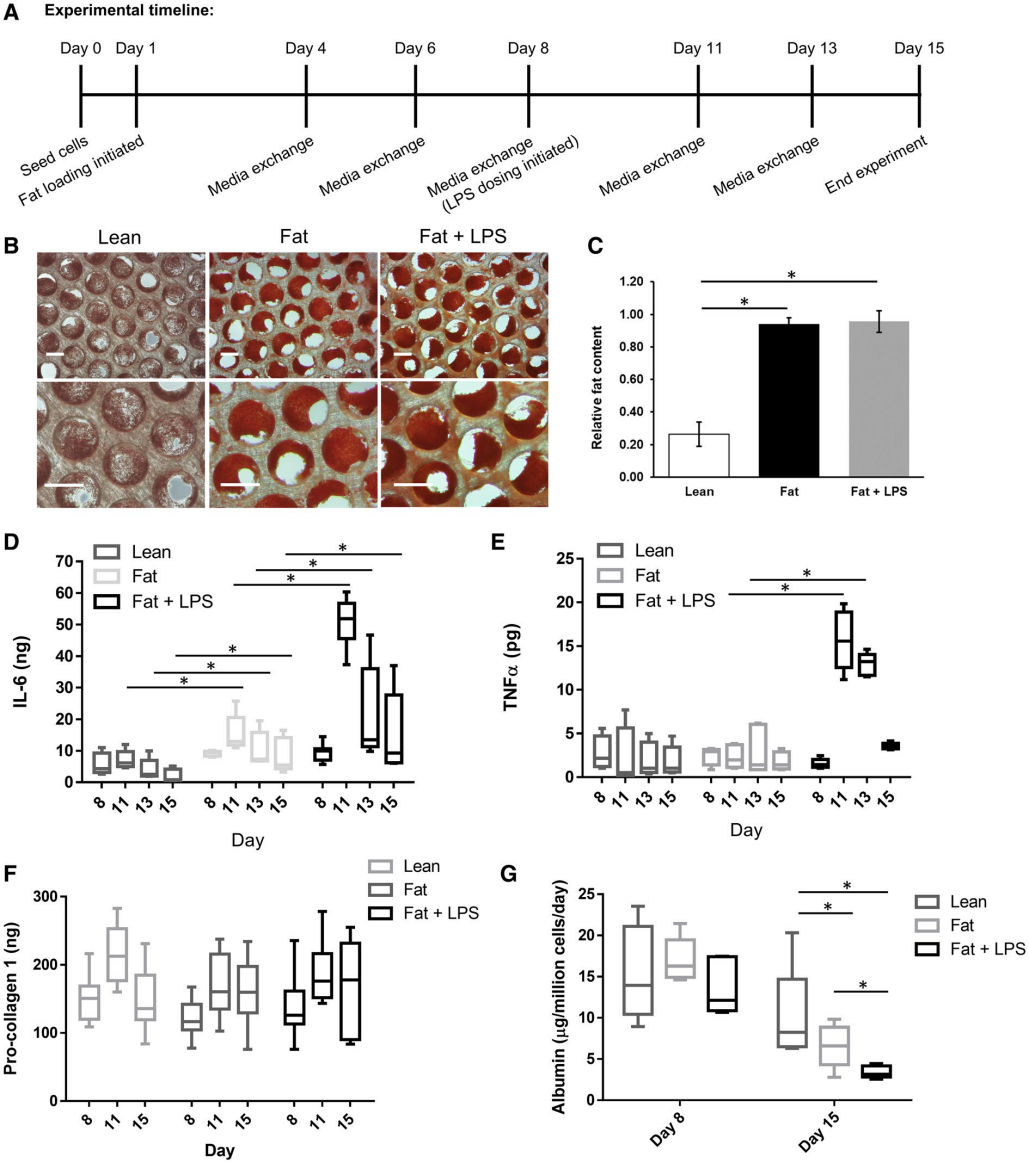
FFAs and LPS together enhance the disease phenotype of the *in vitro* human NASH model. PHH, HK, and HSC co‐cultures (NASH) were cultured in the MPS for 15 days in HEP‐LEAN medium, HEP‐FAT medium, or HEP‐FAT + LPS medium. (A) Fat loading was initiated on day 1, and the addition of LPS was on day 8 following the establishment of the co‐culture microtissues. Fat loading was measured by Oil Red O staining of microtissues, which were observed by microscopy (scaled bar = 200 µm) (B) and quantified by absorbance at 510 nm to give a relative fat content (C). Inflammatory responses were determined by measuring IL‐6 (D) and TNF‐α (E) production by ELISA. (F) The section of procollagen 1 was measured as a fibrosis marker. (G) Cultures were compared for albumin section into the cell culture medium. Data are presented as means ± SD from a minimum of five independent cultures; *P < 0.05.

**Figure 3:**
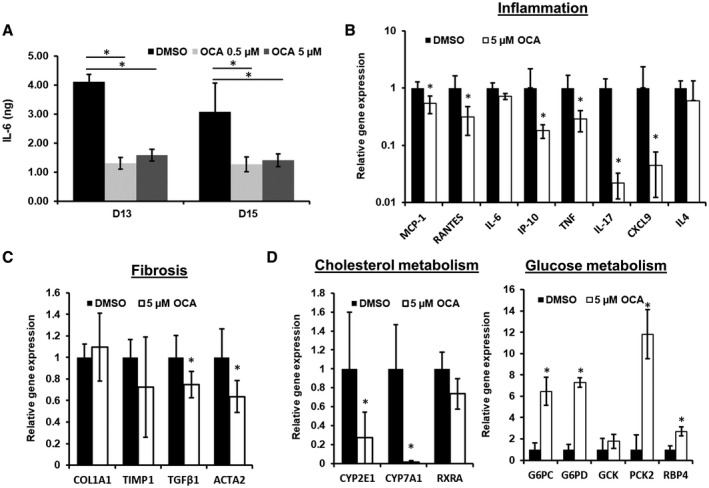
Treatment with OCA reduces the disease phenotype in the MPS NASH model. PHH, HK, and HSC co‐cultures were cultured in the MPS for 15 days in HEP‐FAT medium. On day 8, vehicle (DMSO) or OCA was dosed onto cultures at either 0.5 µM or 5 µM in HEP‐FAT medium. (A) IL‐6 was measured in cell culture medium by ELISA. Relative expression of inflammatory (B), fibrotic (C), metabolic (D) genes associated with NASH following OCA dosing, as measured by quantitative PCR normalized to GAPDH; data are shown as relative expression normalized to the DMSO control. Data are means ± SD from a minimum of three independent cultures; *P < 0.05. Abbreviations: ACTA2, smooth muscle alpha‐2; CYP2E1, Cytochrome P450 Family 2 Subfamily E Member 1; CYP7A1, Cytochrome P450 Family 7 Subfamily A Member 1; G6PC, glucose 6‐phosphatase; G6PD, glucose‐6‐phosphate dehydrogenase; GCK, glucose‐6‐phosphate; PCK2, Phosphoenolpyruvate Carboxykinase 2; RBP4, Retinol Binding Protein 4; RXRA, Retinoid X Receptor Alpha.

Fat loading of hepatocytes is established to generally be the starting point for the development of NASH, but other key factors can play an important role in causing disease progression, including LPS.^31^ Changes to intestinal permeability cause increases in LPS concentrations in the portal vein, which can enhance liver inflammation and disease progression.^32^ We therefore explored how both fat and repeat (chronic) low dosing of LPS play a role in enhancing the disease phenotype within the co‐culture NASH model. PHH, HK, and HSC microtissues were cultured from day 1 in HEP‐LEAN medium (absent of additional FFAs), HEP‐FAT medium or HEP‐FAT + LPS medium, with the LPS dosing starting on day 8 of the culture onward (Fig. 2A). To assess the levels of fat accumulation within the microtissues, scaffolds were stained with Oil Red O, which demonstrated significant intracellular fat accumulation in samples cultured in HEP‐FAT or HEP‐FAT + LPS medium (Fig. 2B,C). The secretion of IL‐6 in the model was enhanced by both fat loading of cells and most significantly by the dosing of LPS (Fig. 2D). Additionally, the production of TNF‐α was also enhanced by LPS dosing (Fig. 2E). However, neither fat nor LPS affected the fibrosis phenotype of the NASH model when assessed through the production of procollagen 1 (Fig. 3F). Finally, it was observed that hepatocytes in the HEP‐FAT and HEP‐FAT + LPS medium produced less albumin than those in the HEP‐LEAN medium (Fig. 3G). Overall, both fat and LPS were found to promote the disease state in the co‐culture NASH model; in particular, both significantly enhance the inflammatory state of the model.

After demonstrating that the co‐culture NASH model recapitulated key aspects of the disease, we explored the effects of OCA on the MPS model. OCA is a semisynthetic bile acid derivative currently in phase III clinical trials for the treatment of NASH, and has been shown to improve histological features of the disease.^33^ The NASH co‐culture model was initially established with HEP‐FAT medium for 8 days and then dosed with OCA in the presence of HEP‐FAT medium, for a further 1 week. OCA was dosed at 0.5 µM and 5 µM, the lower of which equates to the plasma C_max_ of OCA, and the higher dose simulated localized liver C_max_ in patients.^34^

In the co‐culture NASH model, OCA was observed to cause a significant reduction in IL‐6 release when compared with vehicle controls (Fig. 3A). To explore more broadly the effects of OCA dosing, we analyzed the expression of a range of NASH‐associated genes and found that OCA significantly reduced the expression of genes associated with inflammation (e.g., interferon‐inducible protein 10 [IP‐10], monocyte chemoattractant protein 1 [MCP1], TNF‐α) and a small number associated with fibrosis (e.g., TGFβ, ACTA2) (Fig. 3B,C). We also analyzed the expression of a number of metabolic pathway genes and observed genes associated with cholesterol metabolism to be down‐regulated, whereas a number of genes associated with glucose metabolism were up‐regulated (Fig. 3D). Together, these data demonstrate the utility of the *in vitro* NASH model for screening anti‐NASH therapeutics and investigating their mechanisms of action.

The I148M SNP in the *PNPLA3* gene has been strongly associated with NAFLD/NASH and fibrosis development.^3^ Recently, Bruschi et al. demonstrated a direct role of this SNP on the disease phenotype of HSCs.^35^ The authors demonstrated that primary human HSCs carrying the I148M PNPLA3 mutation expressed increased levels of pro‐inflammatory mediators and had higher lipid droplet content.^35^ This study was all performed on isolated monocultures of primary human HSCs or on the human HSC LX‐2 cell line; therefore, we proposed to explore whether I148M PNPLA3 mutant HSCs would influence the phenotype in our *in vitro* co‐culture NASH model.

**Figure 4:**
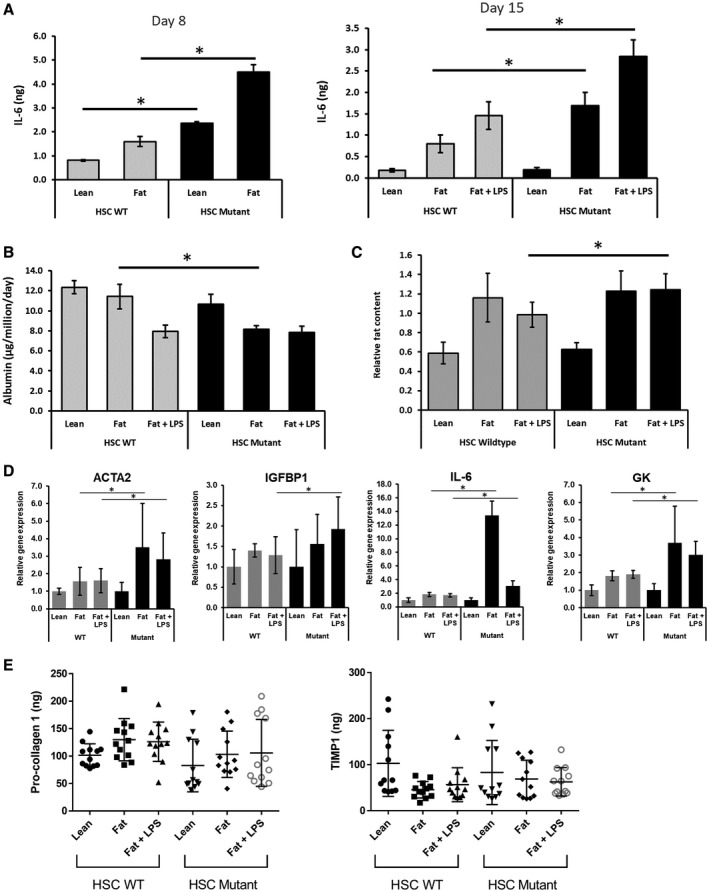
HSCs carrying the PNPLA3 I148M SNP induce a more pro‐inflammatory and steatotic phenotype in the MPS NASH model. PHH, HK, and HSC co‐cultures were cultured in the MPS for 15 days in HEP‐LEAN, HEP‐FAT, or HEP‐FAT + LPS conditions (LPS was added to culture from day 8 onward). Cultures contained either WT HSC or HSC with the PNPLA3 I148M mutation. (A) On day 8 (pre‐LPS dose) and day 15 (post‐LPS dose) of the culture, IL‐6 production was measured by ELISA. On day 15 of the culture, albumin production was measured by ELISA (B), and fat loading was measured by Oil Red O staining of microtissues (C), which was quantified by absorbance at 510 nm to give a relative fat content. (D) Relative expression of total RNA, as measured by quantitative PCR; expression of each gene was normalized to GAPDH and is shown as relative to lean WT samples. (E) Procollagen 1 and TIMP‐1 production was measured by ELISA. Data are presented as means ± SD for 12 independent cultures (3 HSC donors per condition and n = 4 per donor); *P < 0.05. Abbreviations: ACTA2, smooth muscle alpha‐2; GK, Glycerol Kinase; IGFBP1, insulin‐like growth factor‐binding protein 1.

PHH, HK, and HSC co‐cultures were generated with either HSCs genotyped as homozygous wild type (WT) for PNPLA3 (3 biological donors) or homozygous I148M mutant (3 biological donors). The same PHH and HK donors were used throughout the study. The co‐cultures were maintained for 15 days in HEP‐LEAN, HEP‐FAT medium, or HEP‐FAT + LPS medium as previous described (Fig. 4A). We first examined the secretion of the pro‐inflammatory cytokine IL‐6, which was observed to be increased by the presence of the I148M PNPLA3 mutation in the HSCs (Fig. 4A). At both day 8 (pre‐LPS dosing) and at day 15 (post‐LPS dosing), the I148M PNPLA3 mutant HSC induced significantly greater levels of IL‐6 secretion, and these levels were highest in LPS dosed samples (Fig. 4A). The presence of the I148M PNPLA3 mutant HSCs also caused a reduction in the secretion of albumin (Fig. 4B) and induced greater levels of fat accumulation (Fig. 4C). The expression of a number of disease‐related genes, previously observed to be up‐regulated in the co‐culture NASH model (Table 1), were found to be further enhanced by the presence of the PNPLA3 I148M mutant HSC (Fig. 4D). The presence of the I148M PNPLA3 mutant HSC in the co‐culture NASH model, however, did not affect the secretion of profibrotic markers, procollagen 1 and TIMP‐1 (Fig. 4E).

**Figure 5:**
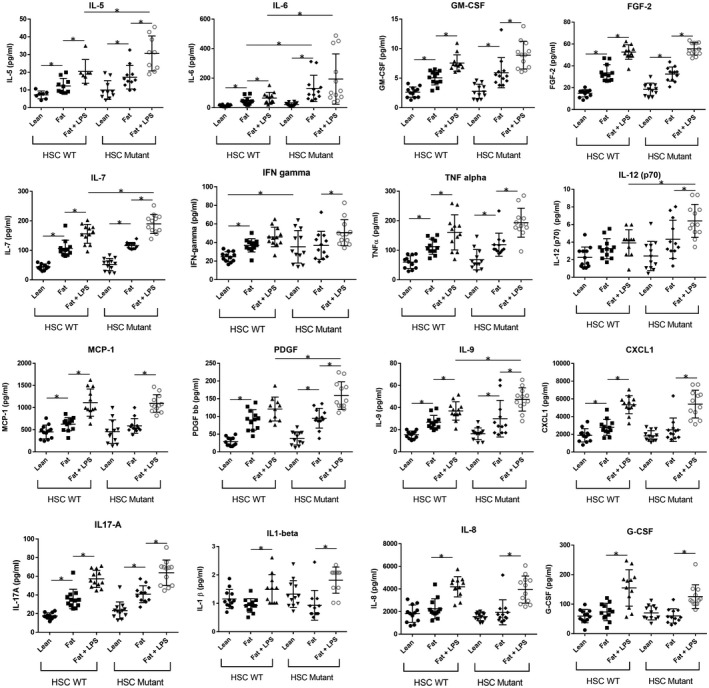
HSCs carrying the PNPLA3 I148M SNP increase the expression of cytokines, chemokines, and growth factors when co‐cultured in the MPS NASH model. PHH, HK, and HSC co‐cultures were cultured in the MPS for 15 days in HEP‐LEAN, HEP‐FAT, or HEP‐FAT + LPS medium (LPS was added to culture from day 8 onward). Cultures contained either WT HSC or HSC with the PNPLA3 I148M SNP mutation. On day 15, cell culture medium was analyzed by Luminex for the presence of human cytokines, chemokines, and growth factors. Data are presented as means ± SD for 12 independent cultures (3 HSC donors per condition and n = 4 per donor); *P < 0.05. Abbreviations: FGF‐2, Fibroblast Growth Factor 2; G‐CSF, Granulocyte‐Colony Stimulating Factor; GM‐CSF, granulocyte‐macrophage colony‐stimulating factor; IFN, interferon; and PDGF, platelet‐derived growth factor.

As the I148M PNPLA3 mutant HSCs had such a predominant effect on the production of IL‐6, we explored how the production of other cytokines and chemokines were affected by the I148M PNPLA3 mutant HSCs (Fig. 5). The secretion of many of these cytokines was shown to be induced by the loading of fat and the additional dosing of LPS (Fig. 5), matching clinical observations (Table 2). When compared with WT HSCs, the I148M PNPLA3 mutant HSC caused increased secretion of cytokines associated with chronic inflammation (e.g., interferon‐γ, IL‐6, IL‐12) and growth factors associated with tissue remodeling and stellate cell activation (e.g., platelet‐derived growth factor, MCP1) (Fig. 5). These data support the observations of Bruschi et al^35^ that primary human HSCs carrying the I148M PNPLA3 mutation have an enhanced inflammatory and activated phenotype. We demonstrate that these PNPLA3 I148M HSCs can promote the NASH disease state in a complex co‐culture disease model.

**Table 2:** Summary of Key Inflammatory Changes in the *In Vitro* NASH Model in Comparison to Clinical Observations

Cytokine	NASH *In Vitro* Model	Clinical Observations
TNF‐α	↑ gene expression (14‐fold increase)	Serum TNF‐α levels correlate with liver disease state
	↑ protein expression (D11 and D13)	
TGF‐β1	↑ gene expression (3‐fold increase)	TGF‐β1 levels increased in patients with NASH
IL‐6	↑ gene expression (160‐fold increase)	Elevated IL‐6 serum concentration in NASH patients
	↑ protein expression (D8 and D15)	
IL‐1β	↑ gene expression (21‐fold increase)	Only expressed in diseased livers and detected in serum of obese patients
	↑ protein expression (when dosing with LPS)	
MCP1 (CCL2)	↑ protein expression (when dosing with fat and fat + LPS)	Elevated CCL2 in NAFLD and NASH patients
IL‐8 (CXCL8)	↔ gene expression unchanged	Higher serum levels of IL‐8 in patients with NASH
	↑ protein expression (when dosing with LPS)	
IL‐1RA	↑ gene expression (2.5‐fold increase)	Increased in human NAFLD locally and systemically

Note:

Clinical observations taken from Braunersreuther et al.^36^ and Niederreiter et al.^37^

Abbreviation: CCL2, C‐C Motif Chemokine Ligand 2.

## Discussion

Understanding the genetic basis and molecular mechanisms that underlie the pathophysiology of NAFLD/NASH will offer the opportunity for the development of new therapies and prevention strategies.^2^ There remains an unmet need for human‐relevant preclinical models that (1) recapitulate key elements of the disease process, (2) facilitate mechanistic study, and (3) allow rapid and efficient evaluation of new therapies. Here we have used a 3D perfused MPS to develop an in vitro human co‐culture model that displayed key aspects of NASH.

To develop our NASH model, we used a liver MPS that has been previously demonstrated to maintain the functions of PHH *in vitro* and prevent rapid dedifferentiation.^22^, ^24^ The system was previously used to develop a model of hepatic steatosis, with PHHs cultured in the presence of FFAs, leading to a range of phenotypic changes that mimicked the earliest stages of NAFLD.^21^ Here we explored the development of a model of a more advanced disease state, through co‐culturing of PHHs with nonparenchymal cells of the liver, namely HKs and HSCs.^2^ To generate a phenotype that mimics NASH, both of the nonparenchymal cells were required, as they contribute to the inflammatory state of the disease and HSCs are the key driver of liver fibrosis. We used an approximately physiological ratio of 10 hepatocytes to every KC and HSC.^27^ As each cell type is individually added to the model, there exists the potential to explore how both the presence and ratio of the different cell types affects the disease phenotype. For reasons of practicality, the three cell types combined to form the co‐culture, namely PHHs, HKs and HSCs, were always derived from three different biological donors. No adverse effects were noted in this regard and a consistent NASH phenotype was observed when different donor material was used. Overall, 9 different HSC donors were used throughout the study (Supporting Table S1). HKs and HSCs were used in the model, as these are associated with initiating early events in NASH development^2^ and are therefore the minimum requirement to achieve a NASH‐like phenotype. Other cell types of the liver are therefore not present in the model (e.g., liver sinusoidal endothelial cells, peripheral immune cells), as these were deemed nonessential to form a NASH phenotype and the addition of further cell types provides challenges of cost, logistics, and reliability for the model. With further development, other cell types could be incorporated into the model to potentially enhance its translational relevance, but while it might be desirable for all cell types of the liver to be included in a model, to date no *in vitro* model exists that incorporates all of the cell types of the liver.^17^ The model additionally does not recapitulate full hepatic zonation, as all of the hepatocytes are exposed to close to atmospheric levels of oxygen, something that is common for *in vitro* models. However, the model does feature an oxygen gradient across the microtissues and has been shown to consume physiologically relevant concentrations of oxygen.^36^

When cultured under fat conditions for a period of 15 days, the co‐cultures demonstrated a phenotype akin to that of advanced NAFLD or NASH, with inflammatory and profibrotic biomarkers detectable in the cell culture medium. Fibrosis biomarkers detected TIMP‐1, procollagen and fibronectin, all of which are well‐established biomarkers of disease development.^28^ A wide range of cytokines were detected in the model, and the expression of many of these was enhanced by the loading of cells with FFAs or additional dosing of LPS (Table 2). Recent review articles have highlighted a key subset of cytokines observed in the clinical disease,^37^, ^38^ and each of these we have detected in our *in vitro* MPS model (Table 2). The co‐cultures also exhibited a range of transcriptomic changes as compared with the PHH‐only steatosis model. Gene‐expression changes associated with NAFLD/NASH development were observed, including increased expression of genes associated with insulin resistance (e.g., IGF1, IGFB1^39^), glucose metabolism (e.g., G6PD,^40^ PDK4^41^), and lipid metabolism (e.g., FABP5,^42^ LPL^43^).

In humans, NAFLD/NASH develops over many years as a result of chronic insults on the liver, whereas rodent preclinical models of the disease are generated over a number of weeks. Previous *in vitro* studies, which are of utility in gaining mechanistic insights, have generally been performed over a small number of days. Here we aimed to develop an *in vitro* NASH model with a duration of a least 2 weeks, to allow an extended window for compound dosing and the potential to observe aspects of disease progression. Thus, the time scale of the model begins to be comparable with rodent models. The *in vitro* NASH model does, however, provide an advantage to rodent models in which 2 weeks is required to generate a disease state, unlike most *in vivo* models that can take weeks or months to establish a NASH‐like phenotype.^6^ This MPS model is also cost‐comparable to genetic and diet‐induced rodent NAFLD models, with the components to generate each 3D co‐culture microtissue costing US $150‐$400, depending on the cell sources used. Whether animal or *in vitro,* the challenge of meaningfully recapitulating a disease that progresses over decades in patients is considerable, and results should be considered with this temporal difference in mind.

We investigated the role of FFAs and the endotoxin LPS on the co‐cultures. We observed that co‐cultures exposed to FFAs had higher levels of intracellular fat accumulation and inflammation (e.g., IL‐6), demonstrating that fat in the model is a driver of the disease phenotype. In the absence of significant inflammation in the monoculture steatosis model, exposure to FFAs was previously reported to increase albumin production due to the over‐fed state of the hepatocytes.^21^ In the NASH model, with the presence of an enhanced inflammatory milieu, albumin levels were observed to slightly reduce over time, correlating with clinical observations, as albumin is a negative acute phase protein.^28^, ^29^, ^30^

LPS is a potential driver of the NASH disease state, as it is proposed to leak from the gut, travel to the liver through the hepatic portal vein, thereby enhancing disease progression, particularly inflammation.^31^, ^44^ Patients with NAFLD have been shown to have increased intestinal permeability, which leads to increased levels of serum endotoxin and TLR‐mediated inflammation.^31^, ^45^ When LPS was repeatedly added to the co‐culture NASH model at low concentrations, to mimic pathophysiological conditions, inflammatory responses were observed to significantly increase. Similar low‐dose LPS studies have been performed in preclinical mouse models^44^ and have shown equivalent responses. Comparing directly to patients is problematic, as determining accurate liver portal LPS concentration is challenging; however, it appears that very small differences in plasma LPS concentration differentiate among healthy patients and patients with steatosis and NASH.^32^ Using LPS in our MPS *in vitro* NASH model demonstrates that a range of disease states can be created in the platform, from simple steatosis (with a PHH monoculture), through to NAFLD and NASH (with a co‐culture) with enhanced inflammation, potentially enabling different aspects of the disease process to be investigated.

To evaluate the pharmacological response of the *in vitro* NASH model, the prototypic anti‐NASH compound OCA, currently being evaluated in phase III trials, was repeat‐dosed for 1 week. Most prominently we observed OCA to reduce a number of inflammatory markers in the NASH model, corroborating the results by other preclinical studies^18^, ^19^, ^46^ and those from phase II clinical trials.^33^ Interestingly we observed a significant decrease in IL‐6 production, but only a minimal effect on gene expression, suggesting that OCA affects the translation, posttranslational modification, or secretion of this cytokine. We observed changes in genes linked to fibrosis and metabolism, including a reduction in the expression of CYP7A1, the rate‐limiting enzyme in the conversion of cholesterol to bile acids.^47^ One reason why OCA appears to be a promising candidate drug is that it affects not only inflammation and fibrosis, but it also affects the metabolic processes associated with NASH.^46^, ^47^ Supporting this idea, we observed up‐regulation of a number of genes associated with glucose metabolism following OCA dosing, including *G6PC*, which is reported to be strongly influenced by OCA dosing in other preclinical models.^9^ The developed co‐culture NASH model should now be explored with a wide range of compounds with varying mechanisms of action to demonstrate its full utility for use in the drug discovery process.

The I148M SNP in the *PNPLA3* gene is highly associated with an increased risk of developing hepatic steatosis, NASH, and liver fibrosis.^3^, ^25^ Recently, a role for the mutated protein has been revealed in HSCs, where it has been shown to cause these cells to increase their activation status, become more pro‐inflammatory, and have a higher lipid content.^35^ By taking HSC donors that contained the WT or mutant I148M PNPLA3 allele, we were able to confirm these findings using our *in vitro* MPS NASH model. The study by Bruschi et al. was performed only on immortalized hepatic cell lines and on monocultures of isolated primary HSCs.^35^ Here we have demonstrated using a more complete *in vitro* recapitulation of the liver, exhibiting a disease phenotype in which the I148M PNPLA3 mutation in HSCs can enhance the overall NASH disease state. In particular, co‐cultures that contained mutant I148M PNPLA3 HSCs produced the highest levels of pro‐inflammatory cytokines and responded most robustly to challenge with LPS. These responses were prominent after 2 weeks in culture, demonstrating the importance of the longitudinal nature of the co‐culture NASH model as opposed to more traditional *in vitro* approaches, which only last a few days. The presence of the I148M PNPLA3 mutant HSCs did not enhance the fibrotic phenotype in the *in vitro* NASH model, and this observation aligns with the findings of Bruschi et al., who also showed the mutant PNPLA3 protein to affect the pro‐inflammatory and steatotic state of HSCs.^35^ The I148M PNPLA3 mutation had the most profound effects in the presence of LPS challenge, suggesting a possible combinatorial effect between leaky gut (leading to LPS release) and the I148M PNPLA3 mutation in patients as two major risk factors promoting NAFLD/NASH progression.

Studying the I148M PNPLA3 mutation here demonstrates how the *in vitro* NASH model is well‐suited to exploring the molecular and genetic basis of the disease. Different cell types and cells with different genetic background, either sourced from donors, as in this study, or modified *ex vivo*, can be combined in a modular fashion to investigate the effects on disease phenotype. This type of study is difficult and time‐consuming in traditional rodent preclinical models. Using this co‐culture NASH model, specific mechanistic insights can potentially be gained in a way that was previously not possible.

In conclusion, we demonstrate that the prolonged co‐culture of PHHs, HKs, and HSCs in a perfused MPS, exposed to FFAs, generates hepatic microtissues that mimic many of the observed features of NASH. The 3D perfused nature of the model allows the co‐cultures to be maintained in a highly functional state for at least 2 weeks, allowing longitudinal changes that approximate disease progression to be explored. The modular nature of the setup allows for specific cell types, with specific genetic backgrounds, to be used, allowing investigation of a range of molecular pathways known to drive the disease. In particular, we demonstrate that the I148M mutation in PNPLA3 can alter the phenotype of HSCs, and this alone can help to enhance the NASH disease state. The *in vitro* co‐culture MPS NASH model, described here, is well‐suited as a tool for drug discovery, to be used either in the exploration of basic biology or in the screening of anti‐NASH compounds.

## References

[1] Pappachan JM , Babu S , Krishnan B , Ravindran NC . Non‐alcoholic fatty liver disease: a clinical update. J Clin Transl Hepatol 2017;5:384‐393. 29226105 10.14218/JCTH.2017.00013 PMC5719196

[2] Friedman SL , Neuschwander‐Tetri BA , Rinella M , Sanyal AJ . Mechanisms of NAFLD development and therapeutic strategies. Nat Med 2018;24:908‐922. 29967350 10.1038/s41591-018-0104-9 PMC6553468

[3] **Romeo** S , **Kozlitina** J , Xing C , Pertsemlidis A , Cox D , Pennacchio LA , et al. Genetic variation in PNPLA3 confers susceptibility to nonalcoholic fatty liver disease. Nat Genet 2008;40:1461‐1465. 18820647 10.1038/ng.257 PMC2597056

[4] Liu YL , Patman GL , Leathart JB , Piguet AC , Burt AD , Dufour JF , et al. Carriage of the PNPLA3 rs738409 C >G polymorphism confers an increased risk of non‐alcoholic fatty liver disease associated hepatocellular carcinoma. J Hepatol 2014;61:75‐81. 24607626 10.1016/j.jhep.2014.02.030

[5] Kohli R , Feldstein AE . NASH animal models: Are we there yet? J Hepatol 2011;55:941‐943. 21708199 10.1016/j.jhep.2011.04.010

[6] Santhekadur PK , Kumar DP , Sanyal AJ . Preclinical models of non‐alcoholic fatty liver disease. J Hepatol 2018;68:230‐237. 29128391 10.1016/j.jhep.2017.10.031 PMC5775040

[7] Sanches SC , Ramalho LN , Augusto MJ , da Silva DM , Ramalho FS . Nonalcoholic steatohepatitis: a search for factual animal models. Biomed Res Int 2015;2015:574832. 26064924 10.1155/2015/574832 PMC4433658

[8] Teufel A , Itzel T , Erhart W , Brosch M , Wang XY , Kim YO , et al. Comparison of gene expression patterns between mouse models of nonalcoholic fatty liver disease and liver tissues from patients. Gastroenterology 2016;151:513‐525. 27318147 10.1053/j.gastro.2016.05.051

[9] Ijssennagger N , Janssen AWF , Milona A , Ramos Pittol JM , Hollman DAA , Mokry M , et al. Gene expression profiling in human precision cut liver slices in response to the FXR agonist obeticholic acid. J Hepatol 2016;64:1158‐1166. 26812075 10.1016/j.jhep.2016.01.016

[10] Janssen AW , Betzel B , Stoopen G , Berends FJ , Janssen IM , Peijnenburg AA , et al. The impact of PPARalpha activation on whole genome gene expression in human precision cut liver slices. BMC Genom 2015;16:1‐13. 10.1186/s12864-015-1969-3 PMC4599789 26449539

[11] Cui W , Chen SL , Hu KQ . Quantification and mechanisms of oleic acid‐induced steatosis in HepG2 cells. Am J Transl Res 2010;2:95‐104. 20182586 PMC2826826

[12] Chavez‐Tapia NC , Rosso N , Tiribelli C . Effect of intracellular lipid accumulation in a new model of non‐alcoholic fatty liver disease. BMC Gastroenterol 2012;12:1‐10. 22380754 10.1186/1471-230X-12-20 PMC3313845

[13] Janorkar AV , Harris LM , Murphey BS , Sowell BL . Use of three‐dimensional spheroids of hepatocyte‐derived reporter cells to study the effects of intracellular fat accumulation and subsequent cytokine exposure. Biotechnol Bioeng 2011;108:1171‐1180. 21449029 10.1002/bit.23025

[14] Gomez‐Lechon MJ , Donato MT , Martinez‐Romero A , Jimenez N , Castell JV , O'Connor JE . A human hepatocellular in vitro model to investigate steatosis. Chem Biol Interact 2007;165:106‐116. 17188672 10.1016/j.cbi.2006.11.004

[15] Joshi‐Barve S , Barve SS , Amancherla K , Gobejishvili L , Hill D , Cave M , et al. Palmitic acid induces production of proinflammatory cytokine interleukin‐8 from hepatocytes. Hepatology 2007;46:823‐830. 17680645 10.1002/hep.21752

[16] Ling J , Lewis J , Douglas D , Kneteman NM , Vance DE . Characterization of lipid and lipoprotein metabolism in primary human hepatocytes. Biochim Biophys Acta 2013;1831:387‐397. 22951416 10.1016/j.bbalip.2012.08.012

[17] Hughes DJ , Kostrzewski T , Sceats EL . Opportunities and challenges in the wider adoption of liver and interconnected microphysiological systems. Exp Biol Med (Maywood) 2017;242:1593‐1604. 28504617 10.1177/1535370217708976 PMC5661768

[18] Davidson MD , Kukla DA , Khetani SR . Microengineered cultures containing human hepatic stellate cells and hepatocytes for drug development. Integr Biol (Camb) 2017;9:662‐677. 28702667 10.1039/c7ib00027h

[19] Feaver RE , Cole BK , Lawson MJ , Hoang SA , Marukian S , Blackman BR , et al. Development of an in vitro human liver system for interrogating nonalcoholic steatohepatitis. JCI Insight 2016;1:e90954. 27942596 10.1172/jci.insight.90954 PMC5135271

[20] Norona LM , Nguyen DG , Gerber DA , Presnell SC , Mosedale M , Watkins PB . Bioprinted liver provides early insight into the role of Kupffer cells in TGF‐beta1 and methotrexate‐induced fibrogenesis. PLoS One 2019;14:e0208958. 30601836 10.1371/journal.pone.0208958 PMC6314567

[21] Kostrzewski T , Cornforth T , Snow SA , Ouro‐Gnao L , Rowe C , Large EM , et al. Three‐dimensional perfused human in vitro model of non‐alcoholic fatty liver disease. World J Gastroenterol 2017;23:204‐215. 28127194 10.3748/wjg.v23.i2.204 PMC5236500

[22] **Tsamandouras** N , **Kostrzewski** T , Stokes CL , Griffith LG , Hughes DJ , Cirit M . Quantitative assessment of population variability in hepatic drug metabolism using a perfused three‐dimensional human liver microphysiological system. J Pharmacol Exp Ther 2017;360:95‐105. 27760784 10.1124/jpet.116.237495 PMC5193075

[23] Rowe C , Shaeri M , Large E , Cornforth T , Robinson A , Kostrzewski T , et al. Perfused human hepatocyte microtissues identify reactive metabolite‐forming and mitochondria‐perturbing hepatotoxins. Toxicol In Vitro 2018;46:29‐38. 28919358 10.1016/j.tiv.2017.09.012

[24] Ortega‐Prieto AM , Skelton JK , Wai SN , Large E , Lussignol M , Vizcay‐Barrena G , et al. 3D microfluidic liver cultures as a physiological preclinical tool for hepatitis B virus infection. Nat Commun 2018;9:1‐15. 29445209 10.1038/s41467-018-02969-8 PMC5813240

[25] Bruschi FV , Tardelli M , Claudel T , Trauner M . PNPLA3 expression and its impact on the liver: current perspectives. Hepat Med 2017;9:55‐66. 29158695 10.2147/HMER.S125718 PMC5683790

[26] Marrone G , De Chiara F , Bottcher K , Levi A , Dhar D , Longato L , et al. The adenosine monophosphate‐activated protein kinase‐vacuolar adenosine triphosphatase‐pH axis: a key regulator of the profibrogenic phenotype of human hepatic stellate cells. Hepatology 2018;68:1140‐1153. 29663481 10.1002/hep.30029

[27] Ebrahimkhani MR , Neiman JA , Raredon MS , Hughes DJ , Griffith LG . Bioreactor technologies to support liver function in vitro. Adv Drug Deliv Rev 2014;69‐70:132‐157. 10.1016/j.addr.2014.02.011 PMC4144187 24607703

[28] Fitzpatrick E , Dhawan A . Noninvasive biomarkers in non‐alcoholic fatty liver disease: current status and a glimpse of the future. World J Gastroenterol 2014;20:10851‐10863. 25152587 10.3748/wjg.v20.i31.10851 PMC4138464

[29] Angulo P , Hui JM , Marchesini G , Bugianesi E , George J , Farrell GC , et al. The NAFLD fibrosis score: a noninvasive system that identifies liver fibrosis in patients with NAFLD. Hepatology 2007;45:846‐854. 17393509 10.1002/hep.21496

[30] Fierbinteanu‐Braticevici C , Baicus C , Tribus L , Papacocea R . Predictive factors for nonalcoholic steatohepatitis (NASH) in patients with nonalcoholic fatty liver disease (NAFLD). J Gastrointestin Liver Dis 2011;20:153‐159. 21725512

[31] Kirpich IA , Marsano LS , McClain CJ . Gut‐liver axis, nutrition, and non‐alcoholic fatty liver disease. Clin Biochem 2015;48:923‐930. 26151226 10.1016/j.clinbiochem.2015.06.023 PMC4558208

[32] Kitabatake H , Tanaka N , Fujimori N , Komatsu M , Okubo A , Kakegawa K , et al. Association between endotoxemia and histological features of nonalcoholic fatty liver disease. World J Gastroenterol 2017;23:712‐722. 28216979 10.3748/wjg.v23.i4.712 PMC5292346

[33] Neuschwander‐Tetri BA , Loomba R , Sanyal AJ , Lavine JE , Van Natta ML , Abdelmalek MF , et al. Farnesoid X nuclear receptor ligand obeticholic acid for non‐cirrhotic, non‐alcoholic steatohepatitis (FLINT): a multicentre, randomised, placebo‐controlled trial. Lancet 2015;385:956‐965. 25468160 10.1016/S0140-6736(14)61933-4 PMC4447192

[34] Edwards JE , LaCerte C , Peyret T , Gosselin NH , Marier JF , Hofmann AF , et al. Modeling and experimental studies of obeticholic acid exposure and the impact of cirrhosis stage. Clin Transl Sci 2016;9:328‐336. 27743502 10.1111/cts.12421 PMC5351006

[35] Bruschi FV , Claudel T , Tardelli M , Caligiuri A , Stulnig TM , Marra F , et al. The PNPLA3 I148M variant modulates the fibrogenic phenotype of human hepatic stellate cells. Hepatology 2017;65:1875‐1890. 28073161 10.1002/hep.29041

[36] Domansky K , Inman W , Serdy J , Dash A , Lim MH , Griffith LG . Perfused multiwell plate for 3D liver tissue engineering. Lab Chip 2010;10:51‐58. 20024050 10.1039/b913221j PMC3972823

[37] Braunersreuther V , Viviani GL , Mach F , Montecucco F . Role of cytokines and chemokines in non‐alcoholic fatty liver disease. World J Gastroenterol 2012;18:727‐735. 22371632 10.3748/wjg.v18.i8.727 PMC3286135

[38] Niederreiter L , Tilg H . Cytokines and fatty liver diseases. Liver Res 2018;2:14‐20.

[39] Adamek A , Kasprzak A . Insulin‐like growth factor (IGF) system in liver diseases. Int J Mol Sci 2018;19:1‐24. 10.3390/ijms19051308 PMC5983723 29702590

[40] Hecker PA , Mapanga RF , Kimar CP , Ribeiro RF Jr , Brown BH , O'Connell KA , et al. Effects of glucose‐6‐phosphate dehydrogenase deficiency on the metabolic and cardiac responses to obesogenic or high‐fructose diets. Am J Physiol Endocrinol Metab 2012;303:E959‐E972. 22829586 10.1152/ajpendo.00202.2012 PMC3469611

[41] Zhang M , Zhao Y , Li Z , Wang C . Pyruvate dehydrogenase kinase 4 mediates lipogenesis and contributes to the pathogenesis of nonalcoholic steatohepatitis. Biochem Biophys Res Commun 2018;495:582‐586. 29128353 10.1016/j.bbrc.2017.11.054

[42] Westerbacka J , Kolak M , Kiviluoto T , Arkkila P , Siren J , Hamsten A , et al. Genes involved in fatty acid partitioning and binding, lipolysis, monocyte/macrophage recruitment, and inflammation are overexpressed in the human fatty liver of insulin‐resistant subjects. Diabetes 2007;56:2759‐2765. 17704301 10.2337/db07-0156

[43] Pardina E , Baena‐Fustegueras JA , Llamas R , Catalan R , Galard R , Lecube A , et al. Lipoprotein lipase expression in livers of morbidly obese patients could be responsible for liver steatosis. Obes Surg 2009;19:608‐616. 19301078 10.1007/s11695-009-9827-5

[44] Imajo K , Fujita K , Yoneda M , Nozaki Y , Ogawa Y , Shinohara Y , et al. Hyperresponsivity to low‐dose endotoxin during progression to nonalcoholic steatohepatitis is regulated by leptin‐mediated signaling. Cell Metab 2012;16:44‐54. 22768838 10.1016/j.cmet.2012.05.012

[45] Marra F , Svegliati‐Baroni G . Lipotoxicity and the gut‐liver axis in NASH pathogenesis. J Hepatol 2018;68:280‐295. 29154964 10.1016/j.jhep.2017.11.014

[46] Morrison MC , Verschuren L , Salic K , Verheij J , Menke A , Wielinga PY , et al. Obeticholic acid modulates serum metabolites and gene signatures characteristic of human NASH and attenuates inflammation and fibrosis progression in Ldlr−/−.Leiden mice. Hepatol Commun 2018;2:1513‐1532. 30556039 10.1002/hep4.1270 PMC6287481

[47] Cipriani S , Mencarelli A , Palladino G , Fiorucci S . FXR activation reverses insulin resistance and lipid abnormalities and protects against liver steatosis in Zucker (fa/fa) obese rats. J Lipid Res 2010;51:771‐784. 19783811 10.1194/jlr.M001602 PMC2842143

