# Supplementary material for: A Microphysiological System for Studying Nonalcoholic Steatohepatitis
Source: Hepatol Commun. 2019 Nov 13;4(1):77–91. doi: 10.1002/hep4.1450 (PMC6939502; doi:10.1002/hep4.1450)
Supplement: Supplementary file 1 [file HEP4-4-77-s001.docx]

**Supplemental materials**


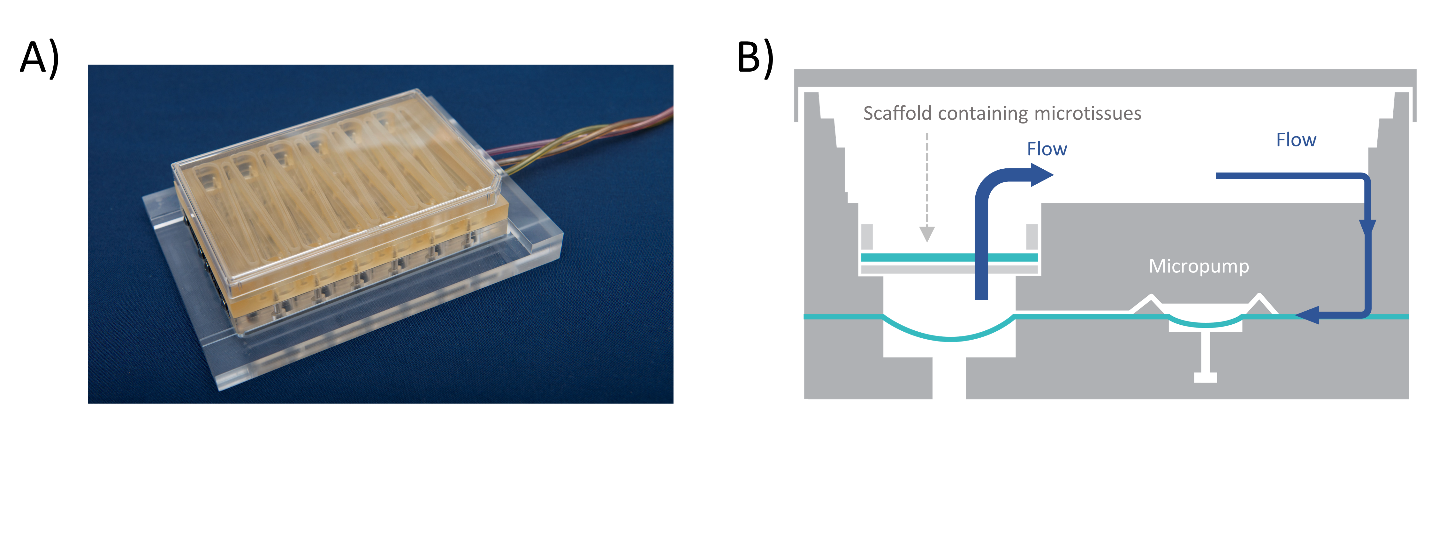


**Supp. Figure 1 – MPS hardware**

A) The perfused MPS cell culture system, utilises open well plates that contain micropumps. Each plate contains 12 fluidically isolated wells. Each well on the MPS plates contains an engineered collagen-coated 3D scaffold which cells are seeded onto to form microtissues. Each scaffold has a depth of 250 µm. B) Schematic representation of a single well on the MPS plate - pneumatically operated micropumps embedded within the plate control the flow of medium within each well. The speed and direction of flow can be adjusted using an electronic controller.


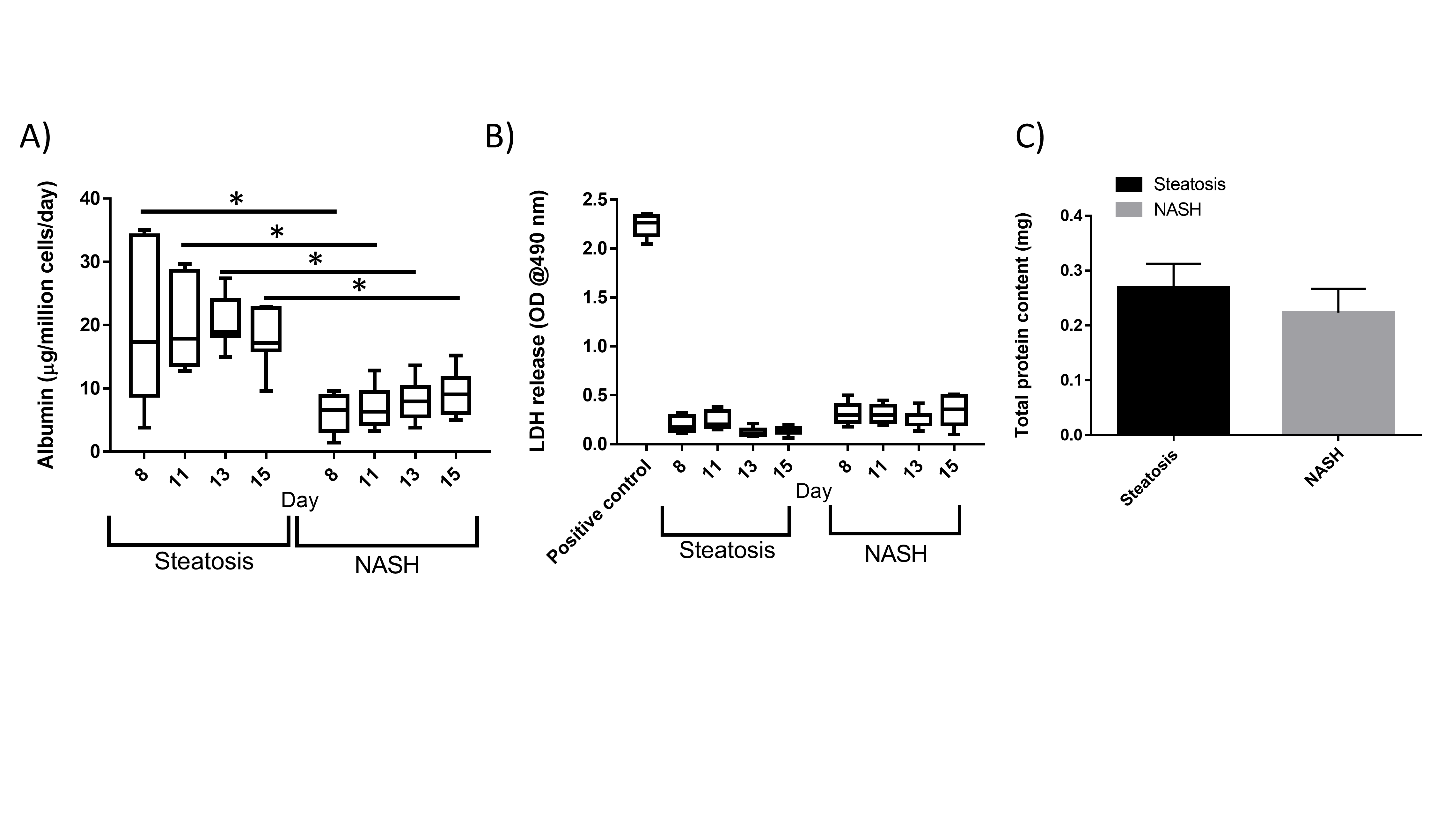


**Supp. Figure 2 – Microtissues in steatosis and NASH models have high cell viability**

PHH alone (Steatosis) or PHH, HK and HSC co-cultures (NASH) were cultured in the MPS for 15 days under high fat conditions (HEP-FAT medium). A) Albumin production was measured in cell culture medium by ELISA. B) LDH release in cell culture medium was measured using the CytoTox96 assay. Positive control is a technical assay control supplier with assay kit. C) Total protein content of 3D cell culture scaffolds was determined by BCA assay. Data are means ± SD from a minimum of six independent cultures; P * < 0.05.


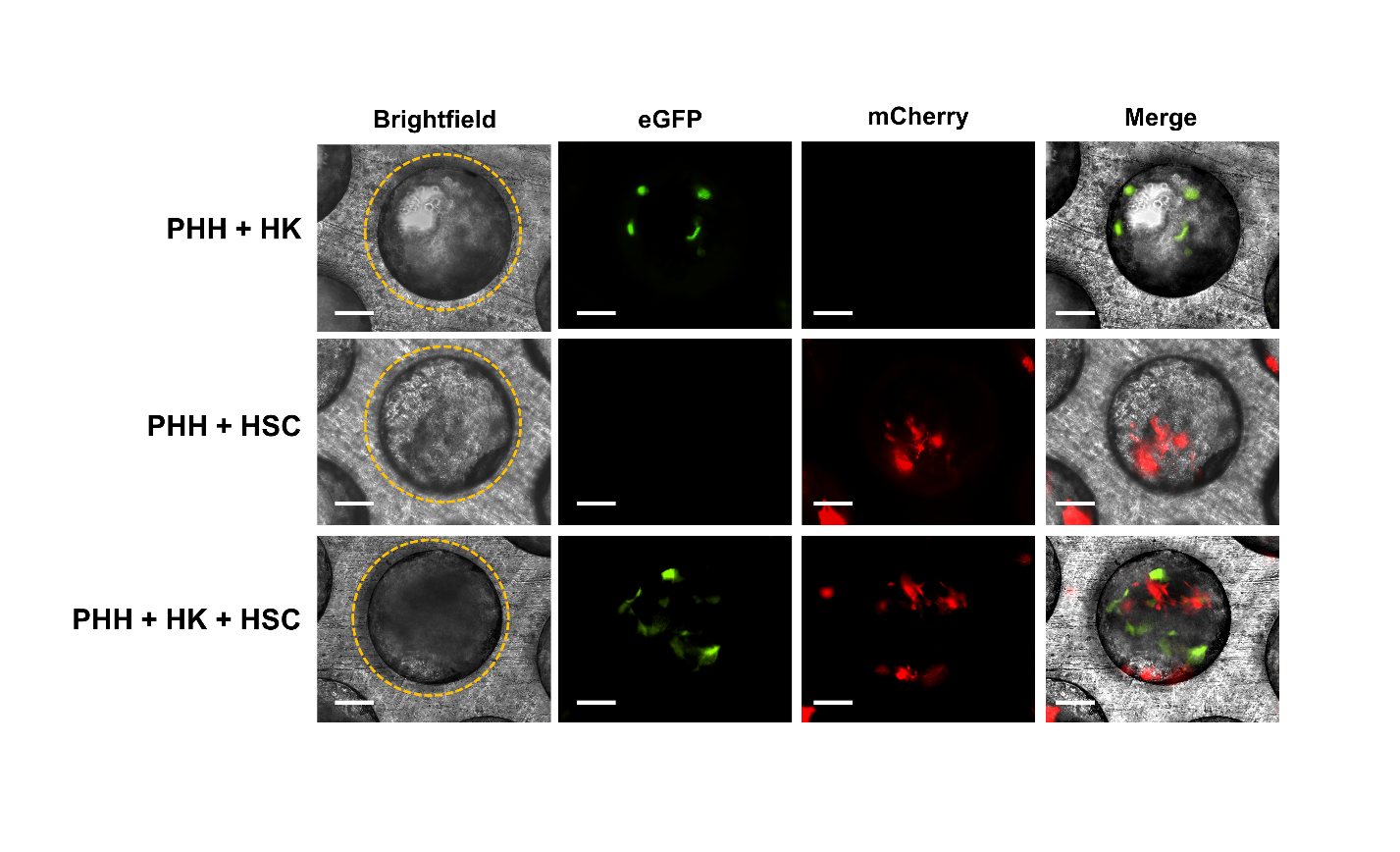


**Supp. Figure 3 – PHH, HK and HSC co-cultures form microtissues within 3D scaffolds in the MPS platform**

To visualise the HK and HSC in the microtissues triple cultures were created of PHH, plus additional primary HK and primary human HSC. All cell types were seeded and cultured for 4 days. Prior to seeding HK and HSC were transduced with adenoviral vectors expressing eGFP and mCherry respectively. The presence of non-parenchymal cells was visualised by fluorescent microscopy. Representative photomicrographs are shown and scale bars are 100 µm. Each photomicrograph shows a single microtissue (highlighted with dotted line) on the collagen coated scaffold.


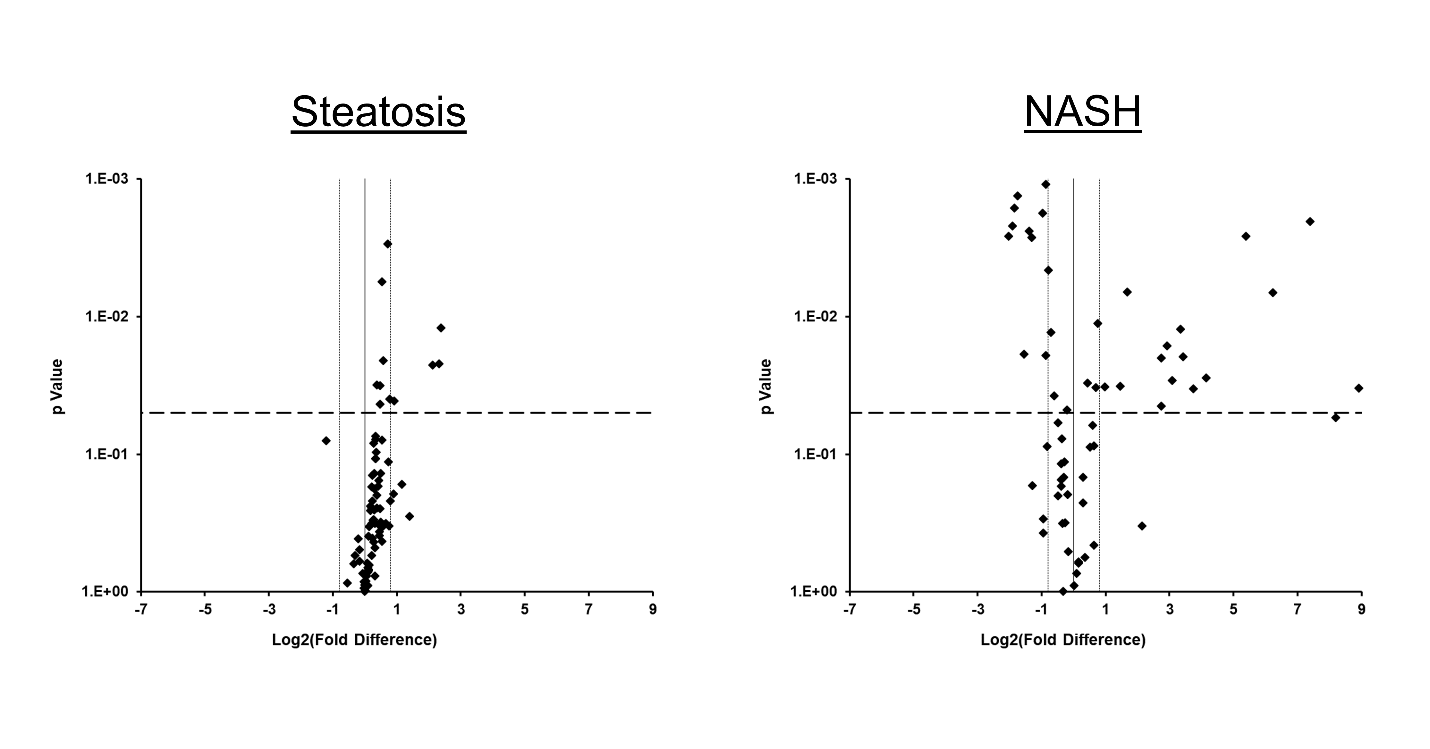


**Supp. Figure 4 – Gene expression profiles are more significantly altered in the co-culture MPS NASH model than in the steatosis model.**

PHH alone (steatosis model) or PHH, HK and HSC co-cultures (NASH model) were cultured in the MPS for 15 days under fat conditions (HEP-FAT medium). Total RNA was extracted and gene expression was compared using Fatty Liver RT2 Profiler PCR Arrays to equivalent samples of PHH cultured for 15 days in HEP-LEAN medium. Gene expression changes were defined by a fold change > 1.75 and P < 0.05. Data is generated from a minimum of three independent cultures per condition.

**Supp. Table 1 – Donor information for primary human Hepatic Stellate cells**

| **Donor ID** | **Sex** | **Conditions** | **PNPLA3 genotype** |
| --- | --- | --- | --- |
| HSC1 | Female | HCC | CG heterozygote |
| HSC2 | Male | Colorectal cancer liver metastasis | CG heterozygote |
| HSC3 | Male | Liver unsuitable for liver transplant | GG homozygous mutant |
| HSC4 | Female | HCC | CC homozygous wild type |
| HSC5 | Female | HCC, hemangioma | CC homozygous wild type |
| HSC6 | Female | Colorectal cancer liver metastasis | CC homozygous wild type |
| HSC7 | Male | Organ unsuitable for transplant | GG homozygous mutant |
| HSC8 | Male | Metastatic NET | GG homozygous mutant |
| HSC9 | Female | HCC | CC homozygous wild type |

**Supplemental experimental procedures**

**Fluorescent microscope imaging of microtissues**

To visualise HK and HSC in the microtissues both cell types were first transduced with adenoviral vectors expressing fluorescent proteins. HSC were transduced with Ad-mCherry (Vector Biolabs) with the virus added directly to cells culturing in a T75 flask at a MOI: 25. The following day cells were trypsinized and washed before seeding into the MPS. HK were transduced with Ad-eGFP (Vector Biolabs) by spinfection. Freshly thawed HK were mixed with Ad-GFP (MOI: 25) and centrifuged at 100 *xg*, 4°C for 50 minutes. Cells were washed and then seeded into the MPS as described above. Following culture scaffolds were fixed for 15 minutes in 4% paraformaldehyde and then washed with PBS before imaging using a Nikon Ti Eclipse fluorescence microscope with FITC and TxRed filters.

**LDH release**

Lactate dehydrogenase (LDH) activity was measured using the CytoTox 96® Non-radioactive cytotoxicity assay (Promega, UK). Fresh cell culture media samples were analysed at each medium change, prior to storage at -80°C. Technical positive control for the assay provided by the manufacture was used for comparison.

**Total protein content of scaffolds**

Total cellular protein was determined for each sample following staining. Each scaffold was washed once in PBS and lysed in 0.1 M NaOH + 2% SDS. Total cellular protein was measured with a Pierce BCA protein assay kit (Thermo Fisher, UK). The relative fat content of each sample was determined by normalising the level of Oil Red O, expressed as absorbance at 515nm to the quantity of total protein.
